# Isolated Hepatic Tuberculosis Discovered Incidentally During Laparotomy for Gastric Perforation in an Immunocompetent Patient: A Case Report

**DOI:** 10.7759/cureus.95481

**Published:** 2025-10-27

**Authors:** Mona Mansouri, Hanane Aksim, Hala Aouroud, Oussama Nacir, Fatimaezzahra Lairani, Adil Ait Errami, Sofia Oubaha, Zouhour Samlani, Khadija Krati

**Affiliations:** 1 Department of Gastroenterology, Mohamed VI University Hospital, Marrakech, MAR; 2 Department of Hepatogastroenterology, Avicenne Military Hospital, Marrakech, MAR

**Keywords:** caseous granuloma, gastric perforation, hepatic tuberculosis, histopathological diagnosis, immunocompetence, liver biopsy, nodular liver lesion, peritonitis

## Abstract

Isolated hepatic tuberculosis is a rare clinical entity, particularly in immunocompetent patients. It is characterized by frequently silent symptoms and nonspecific radiological abnormalities, which complicates its diagnosis in the absence of specific clinical guidance. We present the case of a 39-year-old man with no significant medical history who was admitted to the emergency department with acute peritonitis resulting from gastric perforation. An exploratory laparotomy confirmed the prepyloric perforation and incidentally revealed the presence of two subcapsular hepatic nodules located in segment II. Histopathological analysis of liver biopsies revealed the presence of epithelioid and giant cell granulomatous inflammation, accompanied by caseous necrosis, strongly suggestive of hepatic tuberculosis. The staging assessment, which included a chest CT scan (showing no pulmonary lesions or mediastinal lymphadenopathy), and abdominal imaging, proved negative, reinforcing the diagnosis of isolated hepatic tuberculosis.The patient was placed on standard anti-tuberculosis treatment, with positive clinical results. This case highlights the importance of considering hepatic tuberculosis in the differential diagnosis of hepatic nodular lesions, even in immunocompetent patients, particularly in endemic areas. It also highlights the crucial importance of histopathological analysis in the diagnostic process and in the rapid adjustment of therapeutic management.

## Introduction

Tuberculosis (TB) remains a major infectious disease worldwide, with a high incidence in developing countries [1]. Although the pulmonary form is the most common manifestation, extrapulmonary forms account for approximately 16% of cases and can affect various organs, including, very rarely, the liver [2].

Hepatic TB, particularly in its isolated form, is a rare clinical entity, most often diagnosed in the context of hematogenous dissemination or miliary involvement [3]. It is usually clinically and biologically silent, and radiological abnormalities are nonspecific, making preoperative diagnosis difficult. In immunocompetent individuals, this form remains exceptional and poses a real diagnostic challenge [4]. The incidental discovery of hepatic TB during an emergency laparotomy performed for gastric perforation is an unusual clinical situation, rarely described in the literature.

We report a case of isolated hepatic TB diagnosed incidentally in an immunocompetent patient during surgery for gastric perforation, and propose a review of the clinical, diagnostic, and therapeutic data associated with this rare entity.

## Case presentation

A 39-year-old immunocompetent Moroccan patient, living in an endemic area for TB, was admitted to the emergency department with symptoms of acute peritonitis, including intense and diffuse epigastric pain, accompanied by vomiting and a fever that had persisted for 24 hours. He had no significant medical history and a history of chronic smoking.

On admission, the patient was in poor general condition with a temperature of 38.5°C, tachycardia at 110 beats per minute, and stable blood pressure at 120/75 mmHg. The abdomen was very painful on palpation, with generalized tenderness and maximum sensitivity in the epigastrium. Blumberg's sign was positive, indicating peritoneal irritation. No palpable mass or hepatomegaly was detected on examination. Examination of other systems was unremarkable, with no clinical signs of immunosuppression or pulmonary TB.

Initial blood tests revealed hyperleukocytosis at 15,200/mm³, with a predominance of neutrophils at 85%, indicating an acute inflammatory response. C-reactive protein (CRP) was also elevated at 160 mg/L, confirming the severity of the inflammation. Renal function was preserved, with creatinine at 8 mg/L. Liver function tests showed moderate elevation of transaminases, with aspartate aminotransferase (AST) at 65 IU/L and alanine transaminase (ALT) at 70 IU/L, while alkaline phosphatase and gamma-glutamyl transferase remained within normal limits. In addition, the patient had mild normochromic and normocytic anemia, with hemoglobin at 11.8 g/dL (Table 1). Coagulation tests revealed no abnormalities, and the HIV test was negative.

**Table 1:** Laboratory findings

Parameters	Parameters	Reference range
White blood cell count (/uL)	15,200	4000-11,000
Eosinophilic count (/uL)	140	20-630
Lymphocytic count (/uL)	2600	1000-4800
Neutrophilic count (/uL)	12,260	1400-7700
Hemoglobin (g/dl)	11.8	13-18
Lipase (IU/l)	44	13-78
Alanine aminotransferase (IU/l)	70	<65
Aspartate aminotransferase (IU/l)	65	<50
Alkaline phosphatase (IU/l)	120	40-129
Gamma-glutamyl transferase (IU/l)	51	8-61
Total bilirubinemia (umol/l)	9	<17
Creatinine (umol/l)	8	60-120
Natremia (mmol/l)	135	136-145
Kaliemia (mmol/l)	4.40	3.5-4.6
C-reactive protein (mg/l)	160	<5
Albumin (g/l)	36	35-50

**Figure 1:**
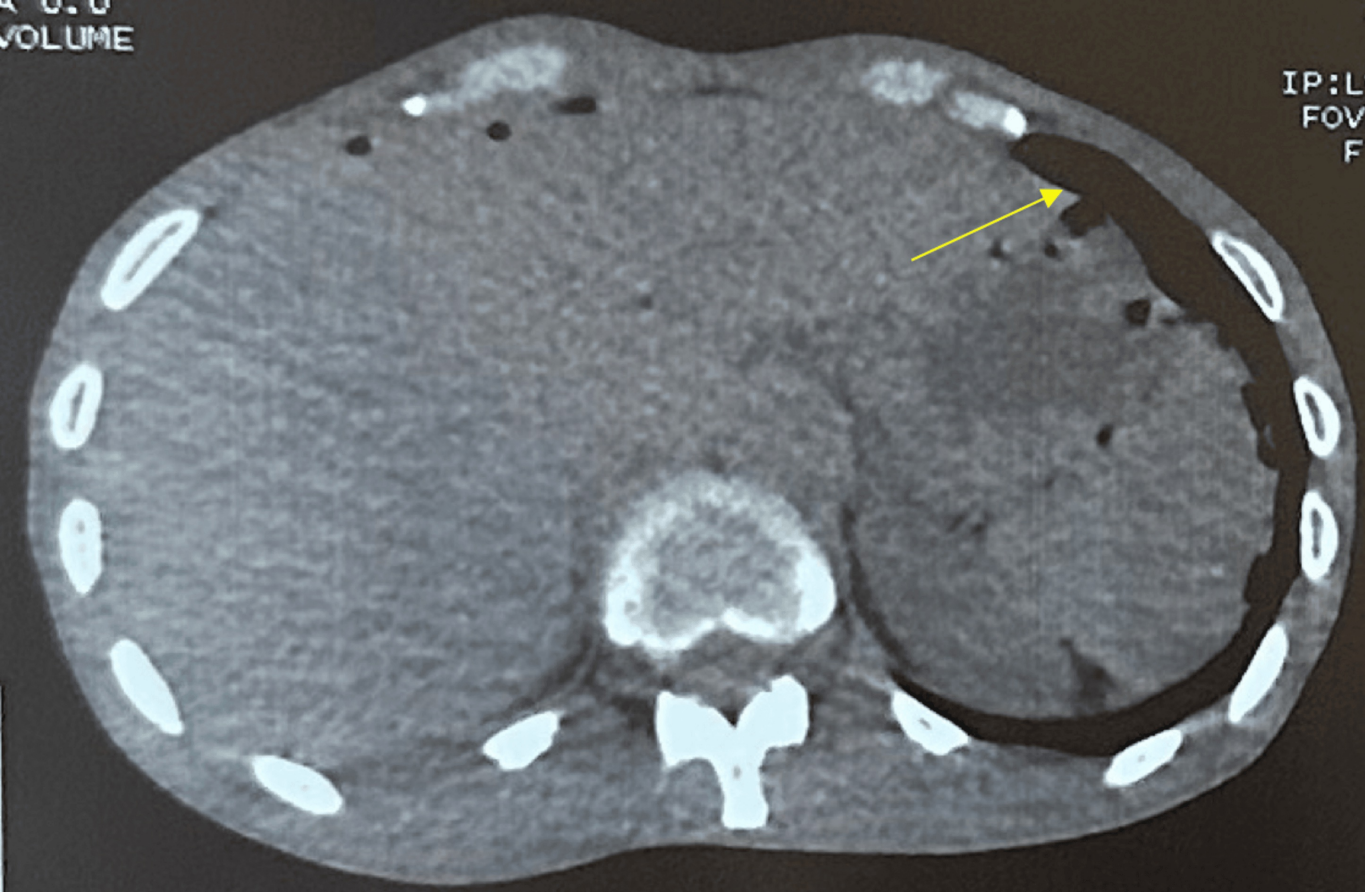
Abdominal CT scan showing pneumoperitoneum bubbles consistent with perforation of a hollow organ (arrow).

Morphologically, an emergency abdominal computed tomography (CT) scan revealed multiple pneumoperitoneum bubbles located in the perihepatic, perigastric, and perisplenic areas, and in the left hypochondrium, associated with mesenteric fat infiltration, predominantly in the supramesocolic region (Figure 1). This finding was highly suggestive of perforation of a hollow organ, most likely the stomach. The liver was of normal size and morphology, with no nodules or focal abnormalities detectable at this stage.

An exploratory laparotomy was indicated as an emergency procedure. Intraoperative exploration revealed a single pre-pyloric gastric perforation, measuring approximately 1 cm in diameter, causing purulent peritonitis. Given the patient’s history of chronic smoking and the absence of any malignant features, the perforation was presumed to result from peptic ulcer disease. The perforation was repaired via primary closure with a Graham omental patch. Incidentally, two subcapsular nodules, measuring 0.25 cm and 0.5 cm, whitish in color and firm in consistency, were observed in segment II of the liver. 

**Figure 2:**
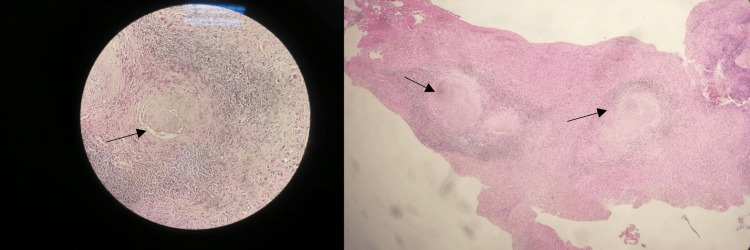
Biopsy of the hepatic nodule showing epithelioid giant cell granulomatous inflammation with caseous necrosis (arrows).

Liver biopsies were performed. Histopathological examination revealed epithelioid and giant cell granulomatous inflammation associated with caseous necrosis, strongly suggestive of TB (Figure 2). The staging assessment, including pulmonary, lymph node, and digestive forms of TB, proved negative, reinforcing the diagnosis of isolated hepatic TB.

**Figure 3:**
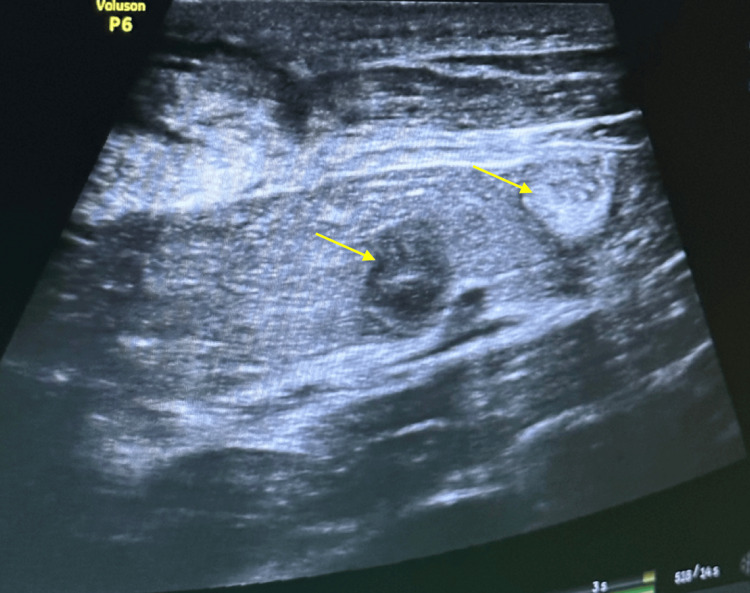
Ultrasound image showing two hypoechoic nodules indicated by yellow arrows in segment II of the liver (arrows).

A postoperative liver ultrasound revealed two hypoechoic nodules located in segment II of the liver (Figure 3).

The patient was placed on anti-TB treatment in accordance with the national protocol, comprising an intensive two-month phase combining four anti-TB drugs (isoniazid, rifampicin, pyrazinamide, and ethambutol), followed by a four-month consolidation phase with isoniazid and rifampicin alone. The total duration of treatment was therefore six months, adjusted according to clinical and radiological progress.

The patient was monitored through regular clinical assessments to check for the disappearance of symptoms such as fever and abdominal pain, as well as through rigorous monitoring of therapeutic tolerance. This included periodic liver and kidney function tests, as well as hematological tests to screen for any treatment-related cytopenias. In addition, radiological examinations were performed several times to assess the regression of liver lesions and rule out any complications.

The clinical course was favorable, with a gradual improvement in the patient's general condition, the disappearance of the feverish syndrome, and the resolution of liver abnormalities on follow-up imaging. At the same time, the patient received therapeutic education to ensure good compliance with anti-TB treatment, which is essential to prevent relapses and the emergence of resistance.

Finally, a family screening consultation was offered as part of a public health strategy aimed at identifying other potential cases among the patient's family and friends.

## Discussion

Isolated hepatic TB in an immunocompetent patient is a rare and confusing clinical form of extrapulmonary TB. It accounts for less than 0.5% of primary TB sites [5]. This rarity can be explained in part by the relative resistance of the liver to TB infection, due to its dual vascularization (portal and hepatic), the phagocytic function of Kupffer cells, and its abundance of lysosomal enzymes [6]. Liver involvement usually occurs as part of hematogenous dissemination during miliary TB or active pulmonary TB [7]. Isolated hepatic involvement, without detectable pulmonary or lymph node foci, could result either from previous dissemination controlled by the host's immunity or from direct implantation that remained latent.

TB remains a major infectious disease worldwide, with a high incidence in developing countries. Morocco, where this case originates, is considered a country with intermediate to high TB prevalence. Although the pulmonary form is the most common manifestation, extrapulmonary forms account for approximately 16% of cases and can affect various organs, more rarely the liver. Including demographic and geographical context underscores the relevance of considering hepatic TB even in immunocompetent individuals [5]. 

The clinical symptoms are often insidious or nonspecific, which greatly complicates diagnosis in the absence of clinical, biological, or radiological clues. In our case, the discovery was entirely incidental, occurring during emergency surgery for peritonitis due to gastric perforation, with no prior clinical suspicion of TB. The biological abnormalities were non-specific, with a slight disturbance in liver function and a marked inflammatory syndrome, which did not allow TB to be distinguished from other infectious or inflammatory causes.

The small hepatic nodules (0.25 cm and 0.5 cm) located in segment II were not detected during preoperative imaging. Macroscopic observation of their whitish, firm, subcapsular appearance prompted a biopsy, which identified, on histopathological analysis, the presence of an epithelioid and giant cell granuloma with caseous necrosis, strongly suggestive of hepatic TB. This diagnosis was made in the absence of other identifiable locations, confirming the isolated nature of the liver involvement.

Imaging has limited value in these subclinical forms. Even CT scanning of the liver may sometimes fail to detect small lesions, or may confuse them with metastases, cysts, or pyogenic abscesses [3]. In such situations, liver biopsy, whether surgical or percutaneous, remains the diagnostic tool of choice. The presence of caseous granulomas allows diagnosis in most cases, although microbiological confirmation techniques (culture, polymerase chain reaction (PCR)) may be useful, particularly in doubtful or atypical forms [7]. 

Similar cases have been reported in the literature. Ayadi et al. describe an immunocompetent patient in whom tuberculous liver nodules were discovered during cholecystectomy, without any preoperative clinical or paraclinical abnormalities [8]. Similarly, Ekanga et al. report a case of primary hepatic TB diagnosed after laparoscopic biopsy in a young immunocompetent adult with chronic hepatic pain [5]. These observations confirm that the clinical picture is often unclear and that diagnosis is based primarily on histology.

The acute presentation of our patient, in the context of gastric perforation, is all the more exceptional. Although digestive TB can rarely manifest as ulcerative perforation, as reported by Amiali et al. [9], the coexistence of prepyloric perforation and tuberculous hepatic nodules suggests either a fortuitous coincidence or possible local or hematogenous dissemination of a paucibacillary form. Intraoperatively, the small, firm, whitish subcapsular liver nodules could have represented a variety of entities, including metastases, small benign tumors such as hemangiomas or adenomas, or parasitic cysts. This highlights the diagnostic challenge faced by the surgeon and reinforces the importance of obtaining tissue for histopathological confirmation. 

Treatment is based on the initiation of multidrug anti-TB therapy according to standard protocols (rifampicin, isoniazid, pyrazinamide ± ethambutol) for a period of six to nine months [7]. The prognosis is generally favorable, as in our case, where a marked clinical improvement was observed after the start of treatment. Surgery is rarely necessary, except in cases of specific complications such as isolated tuberculoma, resistant abscess, or diagnostic uncertainty [10].

Our observation illustrates the need for diagnostic vigilance in the face of any nodular liver lesion, even if discovered incidentally, particularly in areas where TB is endemic. It also highlights the value of systematic intraoperative histopathological examinations when a liver abnormality is visualized, in order to avoid diagnostic delays and adapt therapeutic management.

## Conclusions

Isolated hepatic TB in immunocompetent patients remains a rare and often overlooked condition, which may be discovered incidentally, as in our case. Its nonspecific clinical and biological presentation, combined with radiological signs that are often subtle or inconclusive, makes diagnosis particularly difficult. Histopathology remains the cornerstone of diagnosis, particularly through the identification of granulomas with caseous necrosis. Standard six-month anti-TB treatment is generally effective, although some situations may require additional surgical management. This observation highlights the need to consider hepatic TB as a possibility in the diagnosis of liver lesions, even in non-immunocompromised patients, in order to prevent diagnostic delays and to start appropriate treatment quickly.
